# Mediterranean Diet and Metabolic Syndrome

**DOI:** 10.3390/nu17142364

**Published:** 2025-07-18

**Authors:** Antonia Giacco, Federica Cioffi, Elena Silvestri

**Affiliations:** Department of Science and Technology, University of Sannio, 82100 Benevento, Italy

## 1. Introduction

The Mediterranean diet (MD), a nutritional model rooted in the traditional dietary patterns of Southern Europe and first defined by the pioneering work of Ancel Keys [1], has since been recognized for reducing cardiovascular and metabolic disease risk, as shown in large-scale trials, such as the 2018 reanalysis of PREDIMED [2], and supported by numerous cohort studies [3]. In the context of the rising global prevalence of metabolic syndrome (MetS)—a constellation of interconnected risk factors including central obesity, dyslipidemia, hypertension, and insulin resistance—interest in the MD has been renewed not only for its clinical benefits but also for its capacity to influence physiological systems at the molecular and cellular levels.

This Special Issue was conceived to provide a comprehensive and multidisciplinary examination of the MD’s role in preventing and managing MetS. The aim was to bridge clinical, molecular, and mechanistic research in order to elucidate how this complex dietary pattern may confer its protective effects. This Special Issue brings together contributions ranging from clinical trials and in vivo studies to narrative and systematic reviews that collectively deepen our understanding of how the MD intersects with critical components of metabolic health. In this editorial, we provide an overview of the key findings, themes, and future directions suggested by the six reviews and seven original research articles included in this Special Issue.

## 2. The Mediterranean Diet in the Landscape of MetS Research

A growing body of evidence supports the MD’s capacity to address nearly every hallmark of MetS. Yet despite its well-documented benefits, mechanistic explanations for its broad impact are still evolving. Nutrigenomic and microbial mechanisms are now being unraveled: a 2022 randomized controlled trial demonstrated that a green MD induces gut microbiome changes linked to improved cardiometabolic markers [4], and a 2024 review connected MD adherence with specific microbiota shifts mediating cardiovascular protection [5]. In our call for papers, we emphasized the need to explore not only macronutrient composition but also the bioactive compounds, gut microbiota interactions, hormonal regulation, and intracellular pathways modulated by MD components. The works in this Special Issue reflect this holistic and integrative perspective.

## 3. Integrative Insights from the Reviews

The six reviews in this Special Issue offer complementary perspectives on how the MD exerts its influence on metabolic health.

Mercurio et al. (Contributor 1) provide a thorough narrative review focused on the role of mitochondria as central mediators in non-alcoholic fatty liver disease (NAFLD) and broader metabolic dysfunction. They argue that the MD, through its antioxidant and anti-inflammatory compounds, enhances mitochondrial efficiency, mitophagy, and biogenesis. Despite this promising evidence, they note a major gap: most studies examine isolated bioactive molecules rather than the MD as a synergistic whole. They call for integrative research using systems biology and -omics approaches.

Zupo et al. (Contributor 2) conduct a systematic review of 16 studies linking MD adherence to reduced arterial stiffness—an emerging biomarker of cardiovascular aging and risk. They found consistent inverse associations between MD and pulse wave velocity (PWV) and the Augmentation Index (AIx), although the biological pathways mediating these effects remain incompletely understood.

Scaglione et al. (Contributor 3) examine how MD modulates each component of MetS, from central adiposity and dyslipidemia to blood glucose and blood pressure. Their review underscores the importance of the MD as a “first-line strategy” in the management of cardiometabolic risk, owing to its rich profile of polyphenols, fiber, and healthy fats.

The review by Oteri et al. (Contributor 4) takes a less conventional but highly relevant angle by exploring sexual dysfunction—a frequent but often overlooked comorbidity in MetS. They summarize evidence suggesting that MD improves erectile function and sperm quality in men and sexual health in women through improved vascular function and reduced oxidative stress, highlighting the broader quality-of-life benefits of dietary interventions.

Singar et al. (Contributor 5) focus on almonds, a key component of the MD, and their dual role in cardiovascular and gut health. The review introduces the “gut–heart axis” and emphasizes how almonds, rich in unsaturated fats and polyphenols, improve lipid profiles while modulating gut microbiota toward increased butyrate production—linking metabolic and microbial pathways.

Tsiavos et al. (Contributor 6) present a systematic review on gut microbiota diversity in hypertension, revealing reduced alpha diversity and elevated Firmicutes-to-Bacteroidetes ratios in hypertensive individuals. While not MD-specific, this review underscores the microbial dimension of cardiometabolic risk and points toward future nutritional interventions targeting dysbiosis.

## 4. Contributions from Original Research

In addition to the reviews, this Special Issue features seven original research articles offering diverse insights into how adherence to the MD influences metabolic health across the lifespan, from childhood to old age, and across clinical and community settings.

Two studies focused on pediatric populations. Massini et al. (Contributor 7) evaluated children with primary hyperlipidemia and demonstrated that even modest improvements in adherence to the MD, measured by an updated KIDMED score, significantly reduced LDL and non-HDL cholesterol levels, emphasizing the importance of early dietary intervention to manage cardiometabolic risk. Similarly, Blancas Sánchez et al. (Contributor 8) investigated epicardial adipose tissue (EAT) thickness in Spanish children and adolescents, identifying strong associations between elevated EAT and MetS parameters, including high BMI, LDL cholesterol, and blood pressure—highlighting the utility of EAT as a potential early marker of cardiovascular risk in pediatric populations.

Two clinical trials explored the effects of combining the MD with other lifestyle strategies. Suárez-Cuenca et al. (Contributor 9) conducted a randomized trial on patients with MetS and found that a Mediterranean-style diet, alone or combined with isokinetic exercise, selectively modulated inflammatory cytokines, such as resistin and adiponectin, while body composition changes were modest. Pérez-Vega et al. (Contributor 10) tested the impact of sourdough bread fermentation time on inflammation in individuals with metabolic syndrome. Surprisingly, the short-fermentation sourdough bread reduced inflammatory markers, such as PAI-1 and sICAM, more effectively than long-fermentation bread, although no major changes were observed in gut microbiota composition.

The relationship between dietary patterns and oxidative stress was explored in a unique comparative study by Karras et al. (Contributor 11), who evaluated Orthodox nuns adhering to Christian Orthodox fasting (a plant-based, time-restricted form of the MD) versus laywomen following a 16:8 time-restricted eating regimen without Mediterranean features. The nuns showed increased total antioxidant capacity, whereas laywomen had higher glutathione levels, suggesting differential but complementary effects on oxidative balance in vitamin D-deficient, overweight women.

Socioeconomic and lifestyle determinants were explored by Duarte et al. (Contributor 12), who assessed HRQoL and MD adherence among low-income Portuguese citizens receiving government support. While overall adherence to the MD was not directly associated with improved HRQoL, physical activity and educational level were strong positive contributors, pointing to the multifactorial nature of well-being in vulnerable populations.

Finally, Gómez-Sánchez et al. (Contributor 13) provided robust epidemiological data from over 3400 Caucasian adults, confirming that higher adherence to the MD was associated with a lower likelihood of having metabolic syndrome and its individual components. The study also found favorable associations between diet adherence and systolic and diastolic blood pressure, glycemia, triglycerides, and HDL cholesterol, reaffirming the MD as a protective dietary model in large-scale cross-sectional analysis.

Together, these studies highlight the clinical versatility and population-wide relevance of the MD pattern. From biochemical improvements in lipid and inflammatory profiles to structural markers, such as EAT, and psychosocial determinants, such as HRQoL, the evidence reinforces the value of integrating Mediterranean-style eating habits within preventive and therapeutic strategies targeting metabolic syndrome.

## 5. Strengths, Limitations, and Future Directions

This Special Issue stands out for its comprehensive inclusion of both high-level syntheses and diverse original research. The reviews offer a critical reappraisal of the MD’s mechanisms of action, including emerging roles for mitochondria, the gut microbiome, redox signaling, and chrono-nutrition. Meanwhile, the original studies span a broad demographic spectrum—from children with early metabolic risk factors to elderly women and socioeconomically vulnerable populations—and incorporate both observational and interventional designs.

Limitations include the relatively small sample sizes in several trials, the absence of long-term follow-up in most interventions, and the underrepresentation of non-Mediterranean populations, which could affect generalizability. Nonetheless, these studies highlight promising, underexplored areas. Future research should aim to include multi-omic approaches, longitudinal designs, and translational interventions tailored to real-world complexity, particularly in youth, aging populations, and at-risk communities.

## 6. Looking Ahead: The Future of MD Research

The MD continues to evolve from a cultural heritage into a dynamic, evidence-based model of metabolic health promotion. This Special Issue affirms its preventive and therapeutic potential while highlighting the importance of context: dietary patterns are embedded within lifestyles shaped by socioeconomic status, cultural practices, and environmental pressures.

The path forward includes validating biomarkers of adherence and efficacy, refining dietary assessment tools, and integrating personalized nutrition frameworks [6]. Moreover, advancing digital tools, mobile health technologies, and community-based strategies may help bridge the gap between knowledge and practice—particularly for children, families, and underserved populations where early and sustained intervention could yield the greatest benefits [7].

## 7. Conclusions

Collectively, the contributions to this Special Issue offer a nuanced and up-to-date portrait of how the MD intersects with key metabolic processes, from lipid regulation and glycemic control to inflammation and oxidative stress. The studies also reflect increasing efforts to capture its psychosocial, cultural, and environmental dimensions.

While rates of MetS continue to rise globally, the current literature supports MD as a sustainable, adaptable, and biologically plausible strategy for prevention and care [8]. Continued investment in rigorous, inclusive, and innovative research—across the lifespan and across systems—will be essential to fully realize its promise.
